# Association Between Lifestyle Patterns and Abdominal Obesity with Biochemical and Inflammatory Biomarkers in Adolescents with Down Syndrome: The UP&DOWN Study

**DOI:** 10.3390/nu16223884

**Published:** 2024-11-14

**Authors:** Ana Gutierrez-Hervas, Esther Nova, Rocío Izquierdo-Gómez, Óscar L. Veiga, Carmen Padilla, José Castro-Piñero, Ascensión Marcos, Sonia Gómez-Martínez

**Affiliations:** 1 Nursing Department, University of Alicante, 03690 Alicante, Spain; ana.gutierrez@ua.es; 2 Immunonutrition Group, Institute of Food Science, Technology, and Nutrition (ICTAN), CSIC, 28040 Madrid, Spain; enova@ictan.csic.es (E.N.); amarcos@ictan.csic.es (A.M.); 3 GALENO Research Group, Department of Physical Education, Faculty of Education Sciences, University of Cádiz, 11003 Cádiz, Spain; rocio.izquierdo@uca.es (R.I.-G.); carmen.padilla@uca.es (C.P.); jose.castro@uca.es (J.C.-P.); 4 Biomedical Research and Innovation Institute of Cádiz (INiBICA), Research Unit, 11009 Cádiz, Spain; 5 Department of Physical Education, Sport and Human Movement, Faculty of Education, Autonomous University of Madrid, Ciudad Universitaria de Cantoblanco, 28049 Madrid, Spain; oscar.veiga@uam.es

**Keywords:** Down syndrome, adolescents, inflammatory biomarkers, lifestyle patterns

## Abstract

Background/Objectives: The main objective of this study was to examine the association between lifestyle patterns (physical activity, screen and sleep time and diet) and abdominal obesity, and endocrine, metabolic, and immunological biomarkers in adolescents with Down syndrome (DS). Methods: Eighty-three DS adolescents (38.6% girls), aged 11 to 18 years, from the UP&DOWN study were included. Cluster analysis was performed by including the compliance of recommendations of lifestyle variables, such as moderate to vigorous physical activity (MVPA), screen and sleep time and adherence to the Mediterranean diet (AMD). The waist-to-height ratio was used as an indicator of abdominal obesity. Haematological, biochemical and inflammatory biomarkers were analysed. Results: A three-cluster solution was identified: Cluster 1: adolescents with low compliance; Cluster 2: youth with medium compliance; and Cluster 3: adolescents with high compliance. Significant differences in MVPA (*p* = 0.000), screen time (*p* = 0.004), sleep time (*p* = 0.0001), AMD (*p* = 0.000) and abdominal obesity (*p* = 0.003) were found. Clusters 2 and 3 had lower levels of triglycerides and LDL-cholesterol than Cluster 1. Cluster 2, in which all adolescents met the MVPA recommendations, had the lowest levels of galactin 3. Conclusions: Compliance with lifestyle recommendations (PA, screen and sleep time and AMD) and the absence of abdominal obesity seem to be associated with better biochemical and inflammatory values.

## 1. Introduction

Childhood obesity is one of the main public health problems worldwide [1,2]. According to the World Obesity Federation, in 2020, Spain ranked as the twentieth country in the world with the highest prevalence of overweight or obese children and adolescents [3]. Several years ago, the International Association for the Scientific Study of Intellectual Disabilities identified obesity as a priority in individuals with Down syndrome (DS) [4]. In fact, higher levels of fatness are been found in individuals with DS during adolescence and adulthood, in comparison with the general population [5,6], as well as abdominal obesity [7].

This outcome is especially relevant since low-grade systemic inflammation seems to be involved in other health problems that affect this population with higher incidence than in the population without disabilities, including cardiovascular diseases, obesity, diabetes mellitus, Alzheimer’s and immunodeficiency, and even more so, considering that children and adolescents with DS show more health problems than their peers without disabilities [8,9,10].

Moreover, adolescence is usually linked to changes in life habits and the metabolic and psychological functioning of individuals [11]. Fortunately, physical activity (PA), sedentary activities, like screen and sleep time, and dietary habits are key and modifiable behaviours in children and adolescents, and useful in tackling obesity and health issues associated with fat mass accumulation [12,13,14,15].

Low levels of PA and excessive sedentary habits are linked to negative health outcomes in adolescence [16]. Thus, public health recommendations suggest a minimum of 60 min of moderate to vigorous PA (MVPA) every day and minimise the time that adolescents spend being sedentary to improve and maintain a healthy status [16]. There is evidence indicating that low MVPA is associated with insulin resistance, high levels of blood lipid concentrations, blood pressure or inflammatory proteins in children and adolescents [13]. On the other hand, excessive time spent in sedentary behaviours is associated with an increased risk of cardiovascular diseases [17]. However, there is little evidence about sedentary time and PA levels in adolescents with intellectual disabilities [16]. Some authors have shown that adolescents with DS are less active than their peers [18,19]. Therefore, promotion of PA and reduction in excessive weight should be performed, as these factors can reduce metabolic risk factors in adolescents [17].

The use of electronic devices by children and adolescents for long hours every day is detrimental to their normal development and causes physical alterations, such as myopia [20], and behavioural problems, including self-harm and suicidal behaviours [21], sleep disorders and even childhood depression, anxiety and memory problems [22]. In contrast, meeting the screen time recommendations could generate health benefits for people with disabilities [23]. Sleep duration is another key lifestyle factor for adolescents’ health [24]. Sleep time has been previously associated with the immune profile in adolescents [25]. Indeed, there are several studies about sleep duration and quality in children and adolescents with DS, since this population is well known to show sleep problems, such as obstructive sleep apnea or frequent night awakenings, among others [26,27,28].

Some evidence has shown that the presence of metabolic risk factors and obesity during childhood and adolescence predict the development of metabolic disorders during adulthood [29,30]. It is well known that excessive adipose tissue is a source of an inflammatory status, by secreting molecules such as leptin, C3 and C4 complement factors, and cortisol, among others [31,32]. Therefore, with the aim of controlling obesity, keeping an adequate energy balance, as derived from dietary inputs and PA levels, should be strongly recommended for children and adolescents [33]. This recommendation is especially important for DS adolescents since, in previous studies, we have already shown their peculiar metabolic status regarding their relationship between adiposity and inflammatory biomarkers [34].

Although the individual effect of these different lifestyle patterns (PA, screen and sleep time), as well as body composition, on immunological and inflammatory biomarkers, has already been described in the scientific literature, the combined influence of PA, sedentary behaviours, sleep time, diet and obesity on several inflammatory biomarkers is still unknown in adolescents with disabilities, for example, with DS. Therefore, the current study aimed to examine the association between lifestyle patterns (physical activity, screen and sleep time and diet) and abdominal obesity, and endocrine, metabolic and immunological biomarkers in adolescents with DS.

## 2. Materials and Methods

### 2.1. Sample and Study Design

This study was performed under the umbrella of the UP&DOWN study. The UP&DOWN study is a 2-year follow-up study designed to assess the impact over time of PA and sedentary behaviours on health indicators, such as physical fitness, metabolic and cardiovascular disease risk factors, inflammatory biomarkers and mental health, as well as to identify the psycho-environmental and genetic determinants of PA in a Spanish sample of adolescents with and without DS. Detailed information about its design has been published elsewhere [35]. Parents and school supervisors were informed by letter about the nature and purpose of the study, and written informed consent was obtained from them. Before starting the UP&DOWN study, we contacted 22 special education schools, 5 associations and 3 foundations. A total of 10 special education schools, 3 associations and 2 foundations for people with intellectual disabilities in the communities of Madrid and Toledo (Spain) agreed to participate. Recruiting a total of 109 volunteers with DS between 11 and 20 years old. Taking into account that our aim in this substudy was to cover as many adolescents with DS as possible, all participants aged 11 to 18 years with available blood samples and the rest of the variables analysed were included. A total of 83 adolescents with DS and complete data were included in the cluster analysis and 74 in the biomarker analysis. For the present cross-sectional study, baseline data collected between September 2011 and June 2012 in Madrid and Toledo (Spain) were included. The study protocols were approved by the Ethics Committee of Puerta de Hierro Hospital (Madrid, Spain) and the Bioethics Committee of the Spanish National Research Council (Madrid, Spain). This study was performed following the ethical guidelines of the Declaration of Helsinki 1964 (as revised in 2000).

### 2.2. Lifestyles Variables

#### 2.2.1. Physical Activity

PA was measured by the ActiGraph accelerometer models GT1M, GT3X and GT3X+ (Actigraph^TM^, LLC., Fort Walton Beach, FL, USA). Adolescents wore the accelerometer against the lower back for seven consecutive days using an elastic waistband. MVPA was calculated according to HELENA cut-offs, which were used in previous studies with European children and adolescents [36], and two groups of MVPA were created: compliance with MVPA recommendations if the mean of this activity was ≥60 min per day and non-compliance if the mean was <60 min per day [16].

Screen and sleep time were collected by an ad hoc questionnaire of the UP&DOWN study; this questionnaire was completed by the families with DS adolescents.

#### 2.2.2. Screen Time

The questionnaire included questions about screen time use (viewing TV or video and playing games on a console or a computer). The answers were summarised in the variable screen time (screen hours during weekdays × 5 + screen hours during weekend days × 2)/7. The subjects were also classified into two groups: adequate screen time when it was lower than 120 min (2 h) per day and non-adequate screen time when this time was ≥120 min per day [37].

#### 2.2.3. Sleep Time

The questionnaire included questions about the time to go to bed and stand up during weekdays and weekend days. The variables sleep hours on weekdays and weekend days were created and combined to determine the average of sleep hours (sleep hours during weekdays × 5 + sleep hours during weekend days × 2)/7. Adolescents were classified into two groups depending on the mean of sleep hours per day: adequate sleep time if they slept between 8 and 10 h per day [38] and non-adequate sleep time if they slept less than 8 h or more than 10 h per day.

#### 2.2.4. Adherence to the Mediterranean Diet

The KIDMED test was included in the previously mentioned questionnaire. This test evaluates adherence to the Mediterranean diet (AMD) in the following categories: (1) slow adherence (≥3 points), (2) need to improve the adherence (4–7 points) and (3) optimal adherence to MD (≥8 points) [39]. For this cross-sectional study of adolescents in particular, this variable was dichotomised like the rest of the variables included, classifying into good adherence (≥8 points), as the KIDMED classification considers optimal adherence or poor adherence (<8 points), including slow adherence or need to improve adherence according to the KIDMED classification.

#### 2.2.5. Abdominal Obesity

Height was measured by using a telescopic height-measuring instrument (model SECA 220; Hamburg, Germany), waist circumference was measured with a non-elastic tape (SECA 200; SECA, Hamburg, Germany) at the level of the narrowest part of the torso. Waist-to-height ratio (WtHR) was calculated as waist circumference/height and was used as an indicator of abdominal obesity. Adolescents were classified as presenting abdominal obesity when WtHR ≥ 0.50 in girls and WtHR ≥ 0.51 in boys [40].

### 2.3. Haematological, Biochemical and Inflammatory Biomarkers

Fasting blood samples were collected early in the morning. In all cases, blood was extracted from the cubital vein according to the established protocol [35]. Fourteen haematological variables were analysed for this study: red blood cells × 10^6^ mm^3^, haematocrit %, mean corpuscular volume (MCV) fL, Red Cell Blood Distribution Width (RDW) %, haemoglobin g/dL, mean corpuscular haemoglobin (HCM) pg, cellular haemoglobin concentration mean (CHCM) g/dL, leukocytes × 10^3^/mm^3^, lymphocytes × 10^3^/mm^3^, neutrophils × 10^3^/mm^3^, monocytes × 10^3^/mm^3^, basophils × 10^3^/mm^3^, eosinophils × 10^3^/mm^3^ and platelets × 10^3^/mm^3^. Five biochemical variables were analysed: glucose mg/dL, triglycerides mg/dL, total cholesterol mg/dL, HDL-cholesterol mg/dL and LDL-cholesterol mg/dL. And also, eleven key biomarkers involved in the inflammatory process were analysed for this study: C-reactive protein (CRP) mg/L, as well as C3 mg/dL and C4 mg/dL, complement factors by turbidimetry (AU2700 Olympus analyser; Olympus UK Ltd., Watford, UK); IL-6 pg/mL, adiponectin × 10^6^ pg/mL and leptin pg/mL by immunoassay (xMAP Technology) using a kit (5 + 1) plex, as stated in the article by Gutierrez-Hervas et al. [34]; galactin-3 pg/mL by enzyme-linked immunosorbent assay (Omnikine TM Human Galectin-3 ELISA kit; Assay Biotech, Sunnyvale, CA, USA); and cortisol pg/mL by enzyme-linked immunosorbent assay (Arbor Assays kit, Ann Arbor, MI, USA).

### 2.4. Statistical Analyses

The analyses were performed using the IBM SPSS Statistics, v. 29.0 program and the level of significance was set at *p* < 0.05. Study sample characteristics are presented as mean (SD), frequencies and percentages of recommendations’ compliance. The normality of the variables was checked by the Kolmogorov–Smirnov test. Differences between genders were analysed by t test.

To identify subpopulations with similar health-related lifestyle factors including body composition a K-means cluster analysis was used. Only adolescents with DS who had complete data (without missing) for all the lifestyle and abdominal obesity variables included in the cluster analysis (MVPA, screen and sleep time, AMD and abdominal obesity) were considered (n = 83). All these variables were dichotomised in yes/no compliance with health recommendations for children and youth, to standardise the data set before clustering. When adolescents complied with the recommendations established for each of these variables, they were assigned as 1 and when they did not comply with them, either by excess or default, depending on the variable, they were assigned as 0. Therefore, “1” corresponds to a healthy lifestyle where the recommendations for MVPA, screen time, sleep time and AMD for this population group were met. In the case of abdominal obesity, “1” corresponds to the absence of abdominal obesity (WtHR < 0.50 in girls and WtHR < 0.51 in boys). In the first step, different possible cluster solutions were identified and compared to provide information necessary for the following procedure. Based on the health-related lifestyle factors plus abdominal obesity, information patterns (clusters solution) were identified.

The selected cluster solution resulted in three groups: Cluster 1 = 32, Cluster 2 = 10 and Cluster 3 = 41 adolescents.

Values of the haematology, biochemical and inflammatory variables under study (red blood cells, haematocrit, MCV, RDW, haemoglobin, CHM, CHCM, leucocytes, lymphocytes, neutrophils, monocytes, basophils, eosinophils, platelets, glucose, triglycerides, total cholesterol, HDL-cholesterol, LDL-cholesterol, CRP, C3, C4, IL-6, leptin, adiponectin, galactin-3 and cortisol) were compared between the three groups by Kruskal–Wallis and two-by-two differences was carried out using Mann–Whitney tests, both with exact Monte Carlo test, with a significance level α = 0.05. This analysis was performed as an alternative to the General Linear Model due to lack of normality because the result indicated that normality could not be assumed in these variables (in some cases, normality was not found and it was doubtful in others).

## 3. Results

A total of 83 adolescents with DS (aged 15.3 ± 2.4) were included in the cluster analysis, 32 of them girls (38.6%). According to lifestyle variables, the whole of the sample performed 44.3 ± 21.3 min of MVPA per day and only 15 (18.1%) of them met the recommendations of a minimum of 60 min/day of MVPA. Screen time was slightly superior to the recommended upper limit; the mean was 130.5 (78.5 SD) minutes per day and 37 (44.6%) met the recommendation of less than 120 min/day. Regarding sleep time, more than half of the sample, 51 participants (61.4%), accomplished the recommendations for this age, sleeping between 8 and 10 h per day, with a mean of 9.8 (0.7 SD) hours/day. AMD, according to the KIDMED test, was optimum in 43 (51.8%) adolescents, with a mean of 7.8 (1.7 SD) points in the whole sample. Regarding body composition, 26 (31.3%) of the participants did not show abdominal obesity, as assessed by the WtHR measurement.

**Figure 1:**
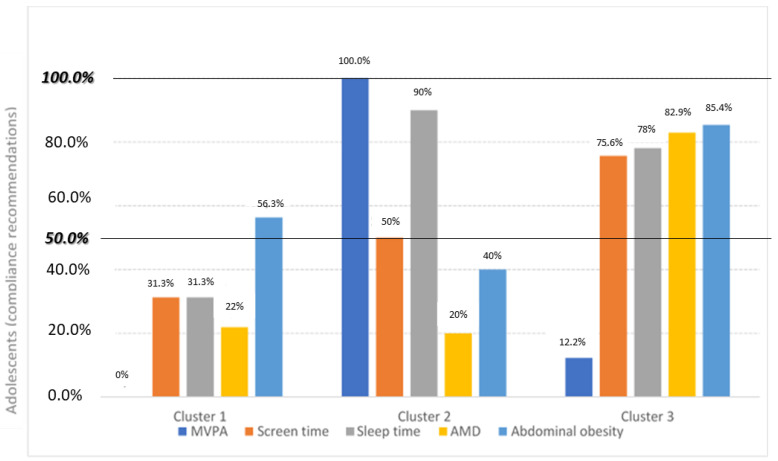
Percentage of DS adolescents who comply with lifestyle recommendations in each of the clusters. Abbreviations: MVPA, moderate-to-vigorous physical activity; AMD, adherence to Mediterranean diet.

**Figure 2:**
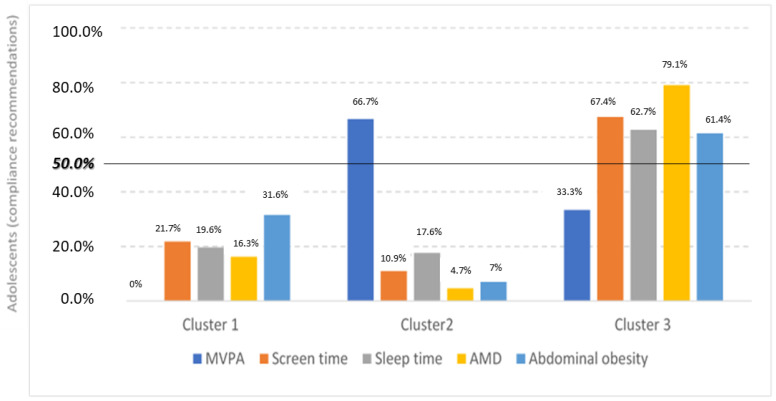
Percentage of adolescents with compliance in lifestyle recommendations by clusters. Abbreviations: MVPA, moderate-to-vigorous physical activity; AMD, adherence to Mediterranean diet.

When we tried to group the adolescents according to their lifestyles plus abdominal obesity, three clusters were statistically generated. Specifically, the clusters were generated based on compliance with the existing recommendations for the lifestyle variables. Compliance with each recommendation was considered when at least half of the adolescents in the cluster complied with the recommendations. Cluster 1, with 32 adolescents, grouped those with generally low compliance with lifestyle recommendations (compliance with abdominal obesity); Cluster 2, the least frequent, with only 10 adolescents, grouped subjects with medium compliance (compliance with screen and sleep time and MVPA); and Cluster 3, including 41 adolescents, mainly grouped those with high compliance (compliance with screen and sleep time, diet and abdominal obesity), as shown in Figure 1 (percentage of adolescents who accomplish with lifestyle recommendations in each of the clusters) and Figure 2 (percentage of adolescents with compliance in lifestyle recommendations by clusters).

As can be seen in the above figures, there are significant differences between all the variables studied (lifestyle and abdominal obesity). There are no significant differences by sex or age among clusters. The values of the lifestyle variables analysed per cluster are shown in Table 1.

The values of the whole blood variables analysed per cluster are shown in Table 2. No differences were found between groups regarding the haematological markers analysed. Concerning the biochemical variables, Clusters 2 and 3 had lower levels of triglycerides than Cluster 1, and LDL-cholesterol in Cluster 1 was higher than in Cluster 3. When analysing inflammatory variables, Cluster 2 reached the lowest levels of galactin 3.

## 4. Discussion

This study provides original and useful information about the relationship between lifestyle behaviours, abdominal obesity and metabolic and inflammatory status in healthy adolescents with DS. Our results show that compliance with lifestyle recommendations (PA, screen and sleep time and AMD) and the absence of abdominal obesity are associated, to some extent, with lower levels of biochemical and inflammatory markers in DS adolescents.

In the current study, less than 20% of the sample accomplished the recommendation of 60 min/day of MVPA; meanwhile, other authors have estimated compliance of 35% in adolescents with DS [41] and 44% in youth without DS from 12 different countries around the world [42].

On the other hand, within sedentary behaviours, screen time takes on special importance because it has been observed that it contributes to the appearance of adverse psychological effects in children and adolescents [43]. In our study, screen time was slightly superior to recommendations in the whole sample, and almost half of the sample complied with the recommendation of less than 2 h per day. Our results are similar to those in other studies performed with DS youth [41]. However, the literature on adults with intellectual disabilities has shown that more than 50% of participants spend four or more hours watching TV or electronic devices [44,45]. Similarly, children and adolescents without DS also report more screen time than recommended around the world [42]. In this sense, there is evidence that an excess of screen time contributes to attention and memory problems during childhood and adolescence [22,43] and DS people have well-known memory problems due to their disability [46].

Another key lifestyle variable was sleep, due to the multiple sleep problems that the DS population suffers during childhood and youth [28] and adulthood [47]. This population used to have sleep problems, such as obstructive sleep apnea, sleep onset difficulties, frequent night awakenings and premature awakening [48,49]. Apnea contributes to a worsening of cognitive function [50] and also can have harmful psychological and physical effects in this population [51]. Furthermore, children with DS frequently experience poor, shorter and disrupted sleep [47] and also may have changes in sleep architecture that affect learning, mood and behaviour [28]. In our results, we have only analysed the hours of sleep per day, and more than half of the sample comply with the recommendations for this age, sleeping more than 8 h per day. Other authors have found a similar sleep time duration to our study, also reported by parents, and have associated the duration and quality of sleep with cognitive problems [27,52]. These authors have tried to understand the relationship between sleep problems and executive functioning in children with DS, which, in turn, have consequences on school behaviour and academic performance [52].

With respect to diet, there is extensive literature about the Mediterranean diet because of its association with health and the prevention and treatment of several pathologies [53,54,55,56,57]. Regarding the AMD in the current study, as assessed by the KIDMED test, it was optimum in half of our DS population. Youth with DS usually consume a high amount of simple carbohydrates, with a low intake of fruits and vegetables, due to the preference for easy-to-swallow foods [58]. Moreover, this population usually has an aversion to certain foods, maybe related to altered perceptions of their consistency, taste, temperature or smell [59]. Our results are significantly higher than those from another study of Italian DS adolescents where the optimal AMD did not reach a quarter of the sample [60]. However, in the population without DS, the AMD in paediatric subjects is generally declining, although varying greatly depending on the country [61]. Spanish children and adolescents without DS usually have a higher AMD than youth from other Mediterranean countries [60,62], and a positive correlation between AMD and health-related quality of life in youth has been observed [63].

During growth and adulthood, DS has been shown to reveal an anthropometric dimorphism associated with trisomy 21, which increases their risk of obesity and abdominal obesity [6,64]. On the other hand, in recent years, obesity has been linked to the shortening of telomeres, which are related to the ageing process, and also in the DS population [65]. According to similar studies, youth with DS show higher rates of overweight and obesity and higher levels of adiposity than their peers without DS [6,66] as well as in adults with DS [64,67]. In the current study, almost 70% of our sample showed abdominal obesity, which may influence their metabolic and inflammatory status. Other authors have reported 21% of abdominal obesity in their Spanish peers without DS [68].

According to PA, studies on DS youth have shown that subjects with lower levels of PA are at higher risk of pathological levels of HDL-cholesterol [60]. In a previous European study assessing youth without DS, we found healthier HDL-cholesterol values together with better glucose metabolism, measured by the HOMA index (homeostatic model assessment) in subjects who practised more PA [69]. Regarding sedentary time, it is also well known that spending excessive and prolonged time in sedentary behaviours, such as screen time, can lead to insulin resistance, vascular dysfunction and increased body fat and blood lipid concentrations in children [70]. In addition, there is also evidence that limited screen time, usually paired with better compliance with MVPA recommendations, has mental health benefits among youth [71].

On the other hand, diet is one of the factors contributing to a higher incidence of overweight and obesity in people with intellectual disabilities, in general, and particularly in DS, including low frequent consumption of healthy foods and poor eating habits [72]. Similarly, in the population with DS, unfavourable diets, such as low AMD, and low PA levels have been seen to be related to obesity [60,64]. For the general population, there is also a relationship between poor diet, low levels of PA and obesity prevalence [73]. In the sample of the current study, the adolescents who showed a high prevalence of abdominal obesity (60%) but met the MVPA recommendations (Cluster 2) reached similar metabolic values than adolescents from Cluster 3, who had a lower prevalence of abdominal obesity (15%), complying with good AMD, but not with MVPA recommendations. This could be due to the anti-inflammatory effect of MVPA, which is useful to limit the negative effects of obesity on youth [74]. It is therefore important to take into account all lifestyles in the health monitoring of these adolescents because of a possible synergy between them.

In regard to immunoregulatory variables, adolescents in Cluster 2, whose subjects performed more MVPA than the others, had lower levels of galactin 3 and a trend towards lower values of other inflammatory molecules. A better state of inflammation markers (cortisol, leptin and C3 and C4 complement factors) was also observed in European youth from different countries who spent more time in MVPA [69]. Other authors have also observed that PA was inversely associated and sedentary time directly associated with circulating levels of CRP [75], leptin, IL-6 and tumour necrosis factor-α (TNF-α) among children without disabilities [76]. We agree with other authors who conclude that exercise (MVPA) is effective as an anti-inflammatory and is useful in limiting the negative effects of obesity on youth. So, it should be considered a key healthy lifestyle factor [74], even in children with systemic inflammatory or autoimmune diseases and other chronic pathologies [77]. Moreover, other research has shown that children and adolescents who spend excessive time in sedentary behaviours, such as screen time, can increase inflammation biomarkers, such as CRP [75], IL-6 and TNF-α [70]. In addition, sleep quality and sleep time during adolescence are inversely associated with CRP levels and a healthier immune profile in children and adolescents without DS [25,78]. In the current study, results emphasise the importance of analysing these lifestyle habit patterns together with body composition in relation to metabolic and inflammatory health in DS adolescents.

The main limitation of the current study is that the sample could not be adjusted by pubertal development because of complications found in the self-reported data collection in the DS group. Another limitation is the cross-sectional design, which does not allow us to draw any conclusions about the causal direction of the associations. In our view, there is a need to explore these causal associations between lifestyle adherence and health biomarkers in future longitudinal studies. However, as a strength, the relatively large sample of children and adolescents with DS and a large number of biochemical biomarkers analysed should be highlighted, as well as the fact that all the relevant lifestyle variables have been assessed.

## 5. Conclusions

A combined effect is observed between compliance to lifestyle recommendations (PA, screen and sleep time and AMD) and the absence of abdominal obesity seems to be associated with better biochemical and inflammatory values, which could have an impact on the health status of adolescents with DS in the future, since they usually suffer from several pathologies associated with their genetic condition, as their characteristics may influence their basic skills and abilities. Even if at these early life stages not many associations were found between compliance with lifestyle patterns, abdominal obesity and the metabolic status markers analysed, some evidence seems to indicate the beneficial effect of MVPA on inflammatory biomarkers. In future studies, it would be interesting to analyse socioeconomic status, family lifestyle or psychological well-being, as they may influence the lifestyle of these adolescents and therefore their metabolic and inflammatory status.

## Figures and Tables

**Table 1:** Main characteristics of the adolescents with Down syndrome according to clusters’ distribution.

Main Characteristics	Cluster 1 n = 32	Cluster 2 n = 10	Cluster 3 n = 41	* *p*-Value
Sex				
Female n (%)	14 (43.8)	4 (40)	14 (34.2)	0.473
Male n (%)	18 (56.2)	6 (60)	27 (65.8)	
	**Mean (SD)**	**Mean (SD)**	**Mean (SD)**	
Age (years)	14.9 (2.1)	15.4 (2.3)	15.6 (2.7)	0.359
MVPA (minutes/day)	34.0 (16.8)	75.5 (15.3)	44.8 (18.2)	**0.000**
Screen time (minutes/day)	162.1 (75.5)	112.7 (72.5)	110.2 (75.7)	**0.004**
Sleep time (hours/day)	10.0 (0.8)	9.2 (0.6)	9.8 (0.5)	**0.001**
AMD	6.9 (1.6)	6.9 (0.7)	8.8 (1.4)	**0.000**
Abdominal obesity (WtHR)	0.51 (0.6)	0.51 (0.7)	0.48 (0.4)	**0.004**

Abbreviations: MVPA, moderate-to-vigorous physical activity; AMD, adherence to Mediterranean diet; WtHR, waist-to-height ratio. * Significant differences among clusters as assayed by Independent-samples Kruskal–Wallis Test. Exact *p*-value of Monte Carlo test. *p* ≤ 0.05.

**Table 2:** Blood variables in DS adolescents according to cluster groups.

Blood Variables	Cluster 1		Cluster 2		Cluster 3		* *p*-Value
	n	Mean (SD)	n	Mean (SD)	n	Mean (SD)	
Red blood × 10^6^ cells/mm^3^	30	4.4 (0.5)	7	4.7 (0.3)	37	4.5 (0.5)	0.349
Haematocrit %	30	42.7 (5.2)	7	45.0 (2.5)	37	43.8 (5.3)	0.330
MCV fL	30	97.2 (4.8)	7	96.0 (3.3)	37	96.7 (4.1)	0.608
RDW %	30	13.8 (1.5)	7	14.3 (1.3)	37	13.7 (1.1)	0.556
Haemoglobin g/dL	30	14.0 (1.7)	7	14.8 (0.9)	37	14.4 (1.844)	0.351
HCM pg	30	31.8 (1.4)	7	31.6 (0.8)	37	31.8 (1.6)	0.912
CHCM g/dL	30	32.7 (1.0)	7	33.0 (1.3)	37	32.9 (1.2)	0.619
Leukocytes × 10^3^ cells/mm^3^	30	4.9 (1.7)	7	5.3 (2.2)	37	5.4 (1.7)	0.209
Lymphocytes × 10^3^ cells/mm^3^	30	1.8 (1.4)	7	1.7 (0.5)	37	2.0 (0.7)	0.120
Neutrophils × 10^3^ cells/mm^3^	30	2.5 (0.9)	7	3.0 (1.8)	37	2.7 (1.2)	0.709
Monocytes × 10^3^ cells/mm^3^	30	0.4 (0.2)	7	0.4 (0.2)	37	0.4 (0.2)	0.537
Basophils × 10^3^ cells/mm^3^	30	0.05 (0.05)	7	0.07 (0.05)	37	0.07 (0.05)	0.230
Eosinophils × 10^3^ cells/mm^3^	30	0.14 (0.14)	7	0.16 (0.16)	37	0.16 (0.17)	0.599
Platelets × 10^3^ cells/mm^3^	30	246.3 (90.4)	7	226.0 (42.8)	37	260.4 (58.1)	0.305
Glucose mg/dL	30	87.2 (8.2)	8	80.9 (9.3)	38	84.5 (8.4)	0.410
Triglycerides mg/dL	30	77.4 (19.7) ^a^	8	56.0 (12) ^b^	38	70.8 (31.7) ^b^	**0.015**
Total Chol mg/dL	30	160.2 (26.4)	8	137.7 (19.9)	38	148.9 (26.8)	0.053
HDL-Chol mg/dL	30	44.1 (6.9)	8	43.1 (10.9)	38	44.9 (9.0)	0.837
LDL-Chol mg/dL	30	100.6 (23.3) ^a^	8	83.3 (14.4) ^a,b^	38	89.9 (22.7) ^b^	**0.048**
CRP mg/L	30	3.62 (9.60)	8	2.99 (4.62)	38	4.16 (9.53)	0.564
C3 mg/dL	30	121.3 (20.4)	8	107.2 (19.0)	38	118.9 (24.4)	0.518
C4 mg/dL	30	30.5 (9.2)	8	27.0 (5.8)	38	28.6 (10.0)	0.395
IL-6 pg/mL	30	38.0 (41.3)	8	28.3 (18.6)	38	38.4 (56.1)	0.719
Leptin × 10^3^ pg/mL	26	11.6 (14.5)	8	5.9 (4)	33	6.4 (6.8)	0.148
Adiponectin × 10^6^ pg/mL	30	10.6 (5.5)	8	12.8 (5.5)	38	12.0 (5.2)	0.335
Galactin 3 × 10^3^ pg/mL	29	2.8 (1.7) ^a^	8	1.2 (758.5) ^b^	36	2.4 (1.5) ^a^	**0.010**
Cortisol × 10^5^ pg/mL	29	1.6 (0.73)	8	1.5 (0.86)	36	1.9 (0.96)	0.120

Abbreviations: MCV, mean corpuscular volume; RDW, Red Cell Blood Distribution Width; HCM, mean corpuscular haemoglobin; CHCM, cellular haemoglobin concentration mean; HCL-Chol, HDL-cholesterol; LDL-Chol, LDL-cholesterol; CRP, C-reactive protein; C3, C3 component factor; C4, C4 component factor. * Significant differences among clusters as assayed by Independent-samples Kruskal–Wallis Test. Exact *p*-value of Monte Carlo test. *p* ≤ 0.05. Different letters (a,b) indicate significant differences between cluster pairs Mann–Whitney test. *p* ≤ 0.05.

## Data Availability

Requestors wishing to access the UP&DOWN trial data used in this study can request to the UP&DOWN trial Steering Committee chair: amarcos@ictan.csic.es. The request will then be passed to members of the UP&DOWN Steering Committee for deliberation.
